# Autotransplantation of teeth with incomplete root formation: a systematic review and meta-analysis

**DOI:** 10.1007/s00784-018-2408-z

**Published:** 2018-03-10

**Authors:** Evelyn C. M. Rohof, Wouter Kerdijk, Johan Jansma, Christos Livas, Yijin Ren

**Affiliations:** 1 Department of Orthodontics, University Medical Center Groningen, Hanzeplein 1 – BB72, NL, 9700 RB Groningen, The Netherlands; 2 Department of Public and Individual Oral Health, Center for Dentistry and Oral Hygiene University of Groningen, University Medical Center Groningen, Groningen, The Netherlands; 3 Department of Oral & Maxillofacial Surgery, University Medical Center Groningen, Groningen, The Netherlands; 4 Private Practice, Zwolle, The Netherlands

**Keywords:** Tooth autotransplantation, Incomplete root formation, Success rate, Survival rate, Systematic review, Meta-analysis

## Abstract

**Objectives:**

The objective of this systematic review and meta-analysis was to determine the rates of survival and success and the complications related to autotransplantation of teeth with incomplete root formation. Additionally, we attempted to identify the prognostic factors that influence the outcome of tooth autotransplantation.

**Materials and methods:**

A literature search for all data published until July 2016 was conducted. Inclusion and exclusion criteria were specified. Risk of bias was assessed with the Newcastle checklist. Meta-analysis was performed by using the DerSimonian-Laird random effect model. The 1-, 5-, and 10-year survival rates and the weighted estimated survival, success, and complication rates per year were calculated.

**Results:**

Thirty-two studies were included for analysis. The survival rates reported after 1, 5, and 10 years were 97.4, 97.8, and 96.3%, respectively. The annual weighted estimated survival rate (98.2%), success rate (96.6%), and complication rates in terms of ankylosis (2.0%), root resorption (2.9%), and pulp necrosis (3.3%) were analyzed. No firm conclusions could be drawn with respect to the prognostic factors due to insufficient evidence of high quality.

**Conclusion:**

The survival and success rates of autotransplantation of teeth with incomplete root formation were high (> 95%), with a low rate of complications (< 5%).

**Clinical relevance:**

Current evidence from the literature on autotransplantation of teeth with incomplete root formation shows favorable survival and success rates and low complication rates, indicating it is a reliable treatment option.

**Electronic supplementary material:**

The online version of this article (10.1007/s00784-018-2408-z) contains supplementary material, which is available to authorized users.

## Introduction

Tooth autotransplantation is a treatment option in cases with tooth loss due to trauma, caries, periodontitis, or endodontic problems and in cases with tooth impaction or agenesis [1–25]. Unlike osseointegrated dental implants, successfully autotransplanted teeth ensure a vital periodontium, continuous eruption, preservation of alveolar bone volume and the interdental papilla, and the possibility of tooth movement by orthodontic or physiological forces [2, 16, 19, 26]. Another advantage of autotransplantation over dental implants is that it can be performed in growing subjects, in whom the incidence of tooth loss due to trauma is relatively high [27, 28]. The longevity and prognosis of autotransplanted teeth are comparable to those of dental implants [29, 30]. However, complications such as inflammatory and replacement root resorption [18, 22, 30, 31], ankylosis [16, 31, 32], pulp necrosis [3–9, 11–15, 33], and compromised periodontal healing [6, 11, 15, 24] may undermine the clinical outcome of tooth autotransplantation.

During the late twentieth century, Andreasen published a series of studies on autotransplantation. In his first study, he reported the standard surgical procedures, which are still being used today [34]. A variety of factors have been suggested to influence the survival and success rates of autotransplanted teeth. Success has been related to patient factors (gender, age) [10, 30, 35, 36], the donor tooth (type, morphology, position, root development) [3, 10, 30, 35, 36], the recipient site (location, local inflammation, alveolar bone volume and quality) [3, 35–37], and the procedure (stabilization method and duration, antibiotic use, damage of the periodontal ligament, need for an autograft or osteotomy, storage method and extraoral time of the graft during surgery, experience of the surgeon, and orthodontic interventions) [3, 30, 35, 36]. Due to the lack of clear evidence to support possible relationships between these factors and eventual success and survival, no firm conclusions can be drawn on the majority of these factors [38].

A recent systematic review and meta-analysis on outcomes of autotransplantation of teeth with *complete* root formation showed very low rates of failure and complications in terms of ankylosis and infection-related root resorption [39]. Nevertheless, endodontic treatment of the transplanted tooth with complete root formation is necessary to prevent or halt the development of periodontal or pulp-related diseases [22, 30, 38]. In contrast, autotransplantation of teeth with *incomplete* root formation offers the advantage of pulp revascularization and reinnervation [30, 34], eliminating the need for endodontic treatment.

Pulp revascularization is closely related to the developmental stage of the transplanted tooth [14, 30]. In another systematic review, the root development stage of the donor tooth was identified as the most important prognostic factor for success of autotransplantation [38]. These authors advised to conduct separate studies and analyses for donor teeth with complete and incomplete root formation.

To date, no systematic review and meta-analysis has been published on the short- and long-term survival rates, the success rates, and the prognostic factors influencing the outcome regarding autotransplantation of teeth with incomplete root formation. Therefore, the aims of this systematic review and meta-analysis were to determine the 1-, 5-, and 10-year and overall survival rates, the overall success rate, and the complication rates of autotransplantation of teeth with incomplete root formation, and to identify the prognostic factors that influence the survival and success.

## Materials and methods

### Protocol development and eligibility criteria

This systematic review is reported according to the PRISMA statement (Appendix A) [40]. The following outcomes were selected: (1) survival rate, (2) success rate, (3) ankylosis rate, (4) root resorption rate, and (5) pulp necrosis rate. The predictors of the outcomes were selected: (6) donor tooth type, (7) recipient site, (8) root development, (9) splinting procedure, (10) splinting duration, (11) orthodontic procedure, and (12) antibiotic regimen.

### Inclusion and exclusion criteria

Inclusion criteria were as follows: human studies; prospective and retrospective studies including randomized clinical trials (RCT), controlled clinical trials (CCT), and case series (CS); involving five or more participants and at least ten permanent transplanted teeth with incomplete root formation; reported or deducible success or survival rates; at least 1-year mean follow-up period.

Exclusion criteria were as follows: application of cryopreservation; replantation after trauma; allotransplantation techniques; osteotomies; case reports, expert opinions, and review articles; animal studies; in vitro studies; publication languages other than English.

### Information sources and literature search

Four electronic databases (PubMed, EMBASE, Web of Science, Cochrane Library) were systematically searched until July 2016, using relevant key words, MESH terms, and synonyms revised for each database (Appendix B). No limitations were applied regarding publication year or publication status. Additionally, a hand search was conducted of the references in the included articles.

Screening and selection were performed independently by two of the authors (E.R. and C.L.). Article titles and abstracts were screened on the selection criteria. When the decision on the basis of title and abstract screening was inconclusive, the full text was acquired. Any disagreement was resolved by discussion and consultation with a third author (Y.R.).

### Quality assessment of included studies

The Cochrane Collaboration’s tool [41] was to be used for assessment for the risk of bias of RCTs. Since only non-RCTs were identified, their methodological quality was assessed with the Newcastle-Ottawa quality assessment scale (NOS) [42]. Two reviewers (E.R., C.L.) independently rated the quality of the included studies and any disagreement was solved by consensus with a third reviewer (Y.R.). The cohort studies could be rated with a maximum of 9 points and the studies were assessed for the following three components according to the NOS: selection, comparability, and outcome. Studies with ≥ 7 points were considered to be of high quality.

### Data extraction

Data was independently extracted by the two authors (E.R., C.L.) within a month period. Only data related to the outcomes of interest were included. Conflicts were resolved by discussion with a third author to reach consensus (Y.R.).

Root development was recorded using the classification as described by Moorrees [43] and used by Andreasen et al. [44] (Table 1). If other classification systems were used in the articles, they were converted to this classification system. Survival was defined as tooth presence during the follow-up. Success was defined as the presence of the tooth in the mouth without ankylosis or inflammatory root resorption, normal mobility, and continuation of root development during the follow-up period. Ankylosis was defined as the absence of clinical mobility with or without root resorption on a radiograph. Root resorption (infection or inflammatory) was defined as the autotransplanted tooth exhibiting resorption signs on a radiograph. However, the data on success, ankylosis, root resorption (infection or inflammatory), and pulp necrosis rates were mainly recorded as indicated in the articles. Authors were contacted for additional data or clarifications when deemed necessary.

**Table 1:** Characteristics and study design of studies included for analysis

Author	Year	Study design	*N*	Age of patients (range)	Donor teeth type (*n*)	Root development^a^ (*n*)	Recipient site	Splinting procedure	Splinting duration (in weeks)	Orthodontics (%)	Follow-up in months (mean)	NOS
Mertens et al. [31]	2014	R	25	17 (10–29)	Md PM2 (10), M3 (15)	3 (21), unknown (4)	Mx I1 (10), Mx PM2 (1), Mx M1 (5), Md PM2 (2), Md M1 (6), Md M2 (1)	Wire and suture	Wire, 6; suture, 2	–	120–240 (−)	4
Nagori et al. [3]	2014a	P	45	–	Mx M3, Md M3	2 (11), 3 (13), 4 (18), 5 (3)	Mx M1, Md M1	Wire or suture	Wire, 2; suture, 1	No	15–24 (20)	6
Nagori et al. [4]	2014b	P	13	–	Mx M3, Md M3	1–2, 2, 3, 4	Mx M1, Mx M2, Md M1, Md M2	Wire or suture	Wire, > 2; suture, 1	–	- (16)	5
de Carvalho et al. [37]	2014	R	21	–	I, C, PM, M2, M3	Mean stage 3	–	–	–	Yes (15%)	6–240 (84)	6
Plakwicz et al. [5]	2013	P	23	13 (10–17)	Mx PM2 (17), Md PM2 (6)	Mean stage 2–3, (range 1–4)	Mx PM2 (2), Md PM2(13), Mx I1 (4), Mx PM2 (1), Md PM2 (3)	Sutures	2	Yes (17%)	6–78 (35)	7
Schütz et al. [6]	2013	R	57	17 (14–21)	Mx M3 (47), Md M3 (10)	2 (12), 3 (26), 4–5 (19)	Mx PM2 (6), Mx M1 (19), Mx M2 (1), Md PM2 (17), Md M1 (12), Md M2 (2)	Wire (86%), orthodontic arch (12%), suture (2%)	5 (2–9)	Yes (12%)	8–64 (26)	5
Shahbazian et al. [33]	2013	P	24	11 (9–18)	PM (22), M (2)	1–2 (1), 2–3 (21), 3–4 (2)	I (11), PM (11), PM/M (2)	Flexible orthodontic wire	–	No	12 (12)	7
Mendoza-Mendoza et al. [7]	2012	R	12	10 (9–13)	PM	2 (4), 3 (7), 4 (3)	Mx I1	Suture	–	Yes (100%)	120–168 (144)	4
Isa-Kara et al. [8]	2011	P	11	–	Mx M3, Md M3	> 2	Mx M1, Md M1, Md M2	Thermoplastic retainer	4	No	31–47 (37)	6
Vilhjálmsson et al. [9]	2010	R	26	–	Mx I1, Mx I2, Mx C, Mx PM, Mx PM, Mx M3	3 (11), 4 (6), 5 (9)	Mx I1, Mx I2, Mx C	–	–	–	1–158 (55)	6
Gonnissen et al. [10]	2010	R	17	–	Mx C, Md C, Md M	2–3 (3), 3–5 (14), unknown (18)	Mx C, Md C	Wire or trauma splint	–	Yes (−)	72–168 (132)	4
Mensink and van Merke-steyn [11]	2010	R	62	–	Mx PM	2–3 (53), 3 (6), unknown (3)	PM	Suture	1	Yes (98%)	12–60 (21)	5
Yan et al. [12]	2010	P	16	–	Md M3	5	M	Wire or suture	1	No	12–132 (62)	6
Díaz et al. [13]	2008	P	10	10 (7–12)	Md PM1 (6), Md PM2 (4)	Open	Mx I1	Composite wire	4 (1–9)	Yes (50%)	5–27 (17)	6
Tanaka et al. [45]	2008	R	19	–	Mx PM, Md PM	2 (2), 3 (17)	Mx I, Mx C, Mx PM, Md PM	–	–	Yes (100%)	48–168 (108)	5
Jonsson and Sigurdsson [14]	2004	R	35	–	Mx PM1, Mx PM2, Md PM1	2–3 (8), 3–4 (21), 4–5 (2), 5 (4)	Mx PM, Md PM2	Suture	1–2	Yes (88%)	29–267 (124)	5
Myrlund et al. [46]	2004	P	68	12 (7–20)	PM	Open	–	–	–	–	48 (48)	5
Bauss et al. [15]	2002	P	76	18 (16–20)	Mx M3 (40); Md M3 (36)	2, 3	Mx PM/M (25), Md PM/M (51)	Wire (45%), suture (55%)	Wire, 4; suture, 1	–	12–73 (41)	7
Czochrowska et al. [16]	2002	R	33	12 (8–15)	Mx I2 (2), Mx PM (10), Md PM (16), supernumerary teeth (2)	Open	Mx I1 (6), Mx I2 (3), Mx C (5), Mx PM (7), Md I (2), Md PM (7)	–	–	Yes (67%)	204–492 (317)	4
Czochrowska et al. [2]	2000	R	45	11 (7–14)	PM	Open	Mx I1 (39), Mx I2 (6)	Suture	1–2	Yes (−)	SD 13 (48)	4
Josefsson et al. [17]	1999	R	99	–	Mx PM1, Mx PM2, Mx M2, Mx M3, Md PM2, Md M3	Open	Md PM2	Suture	1	Yes (47%)	48 (48)	5
Lundberg and Isaksson [18]	1996	R	204	15 (−)	PM (80), M (122)	2, 3	I (6), C (4), PM (158), M (34)	Suture	1	–	6–72 (−)	5
Marcusson and Lilja-Karlander [19]	1996	R	29	–	PM (21), M (8)	Open	PM, M	Suture	1	–	36–192 (−)	5
Kugelberg et al. [20]	1994	R	23	–	Mx I, Mx C, Mx PM1, Md I, Md PM1, Md PM2	3	Mx I1, Mx I2	Suture	1	Yes (−)	12–48 (38)	5
Schatz and Joho [21]	1992	R	40	14 (9–20)	Mx M3 (11), Md M3 (9), PM1 (12), PM2 (8)	1, 2, 3, 4	–	Orthodontic arch	2–6	No	12–136 (64)	7
Kristerson and Lagerstrom [22]	1991	P	41	–	PM (21), M (15), C (2), I (1)	1 (7), 2 (14), 3 (14), 4 (3), 5 (3)	Mx I	Wire or suture	Wire 1–3; suture 1	–	48–204 (90)	5
Andreasen et al. [30]	1990b	P	337	–	Mx PM1, Mx PM2, Md PM1, Md PM2	0 (2), 1 (4), 2 (73), 3 (210), 4 (28), 5 (20)	Mx anterior; Mx PM; Md PM; other	Suture, flexible rigid, or no splinting	–	Yes (46%)	12–156 (−)	6
Andreasen et al. [35]	1990c	P	337	–	Mx PM1, Mx PM2, Md PM1, Md PM2	0 (2), 1 (4), 2 (73), 3 (210), 4 (28), 5 (20)	Mx anterior; Mx PM; Md PM; other	Suture, flexible rigid, or no splinting	–	Yes (46%)	12–156 (−)	6
Hernandez and Cuestas-carnero [23]	1988	P	10	– (13–19)	M3	Mean stage 1	M1	Suture	2	–	36 (36)	6
Kristerson [24]	1985	P	84	–	Mx PM, Md PM	1 (21), 2 (14), 3 (38), 4 (9), 5 (2)	–	Orthodontic arch, suture, or stainless steel wires	1–> 6	–	36–216 (76)	7
Borring-Møller et al. [25]	1979	P	15	17 (15–20)	Mx M3 (6), Md M3 (9)	1, 2, 3	Mx M1 (5), Md M1 (10)	Wire (40%), suture (60%)	Wire, 6; suture, 1	No	3–84 (31)	6
Slagsvold and Bjercke [1]	1974	R	34	12 (8–16)	Mx PM1 (5), Mx PM2(13), Md PM1 (2), Md PM2 (14)	0, 1, 2, 3, 4	Mx PM (24), Md PM (10)	Sutures	–	No	40–166 (74)	4

*P*, prospective study design; *R*, retrospective study design; *Mx*, maxilla; *Md*, mandible; *I*, incisor; *C*, canine; *PM*, premolar; *M*, molar; *NOS*, Newcastle-Ottawa Quality assessment scale, number of points given for selection, comparability, and outcome categories [42]

^a^The stage of root development according to a qualitative classification by Morrees [43]. Stage 1, initial to one quarter root formation; stage 2, one half formation; stage 3, three quarters root formation; stage 4, full root formation with open apical foramen; stage 5, full root formation with half-open apical foramen; stage 6, full root formation with closed apical foramen

### Statistical analysis

Since the follow-up length varied in the included studies, the weighted average rates per year of success and survival were determined in order to compensate for the variability in the reported study durations. This weighted average rate per year was not meant to reflect the actual annual success or survival rates, but to provide clinically relevant indications on the success and survival of the treatment modality of autotransplantation of teeth with incomplete root formation, taking into consideration the full follow-up length of all included studies. Ankylosis, root resorption, and pulp necrosis rates were corrected for study durations in the same way resulting in annual rates. In addition, analyses were performed separately for the different types of donor teeth and different recipient locations. When all articles reported a 100% survival or success rate, no analysis was performed. Articles not providing the mean follow-up were excluded. The weighted average rates per year as well as the weighted average 1-, 5-, and 10-year survival were estimated with a DerSimonian-Laird random effects model [47].

The heterogeneity between studies was analyzed using Cochran’s *Q* test and *I*^2^. Meta-analysis was performed using statistical software package (Comprehensive Meta-Analysis Version 3.3.070, Biostat Englewood, NJ, USA).

## Results

### Study selection

**Fig. 1:**
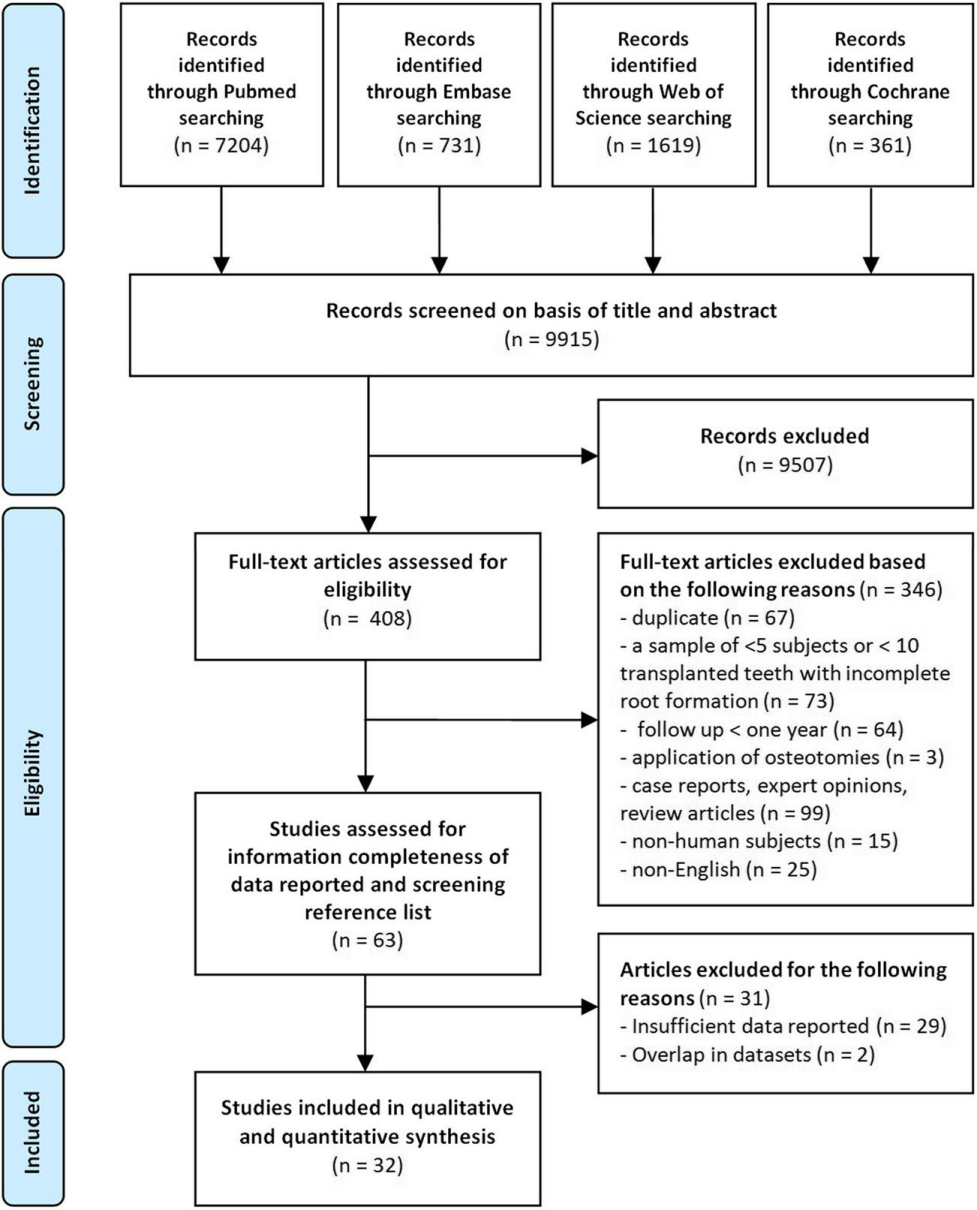
Information through the different phases of a systematic review based on the PRISMA guidelines

The search yielded 9915 articles in total. A detailed overview of the selection process is illustrated in Fig. 1. After screening the titles and abstracts, 408 articles qualified for full text assessment. Subsequently, 63 articles met the inclusion criteria. Hand search of reference lists of the eligible articles resulted in no further additions. Twenty-nine articles were excluded because of not reporting outcomes for autotransplanted teeth with incomplete root resorption separately or not providing sufficient data to answer the research questions. Those studies had therefore methodological inadequacies that could be associated with bias. Finally, 32 articles were considered eligible for qualitative and quantitative analysis (Table 1).

### Study characteristics

Among the 32 included articles, 15 were prospective and 16 were retrospective cohort studies and one case series [7]. Fifteen articles reported results of autotransplantation, solely of teeth with incomplete root formation. The other 17 articles provided data of autotransplantation teeth of both complete and incomplete root formation. We only included data of incomplete root formation in our analysis. Details of the included studies are given in Table 1.

### Quality assessment of included studies

Only five articles could be considered to be of high quality (Table 1; Appendix C). Only one of the studies had blinded recordings of the results [33]. Six studies scored four stars and 11 studies, five stars. These studies had therefore methodological inadequacies that could be associated with bias.

## Primary outcomes

### Survival rate

**Fig. 2:**
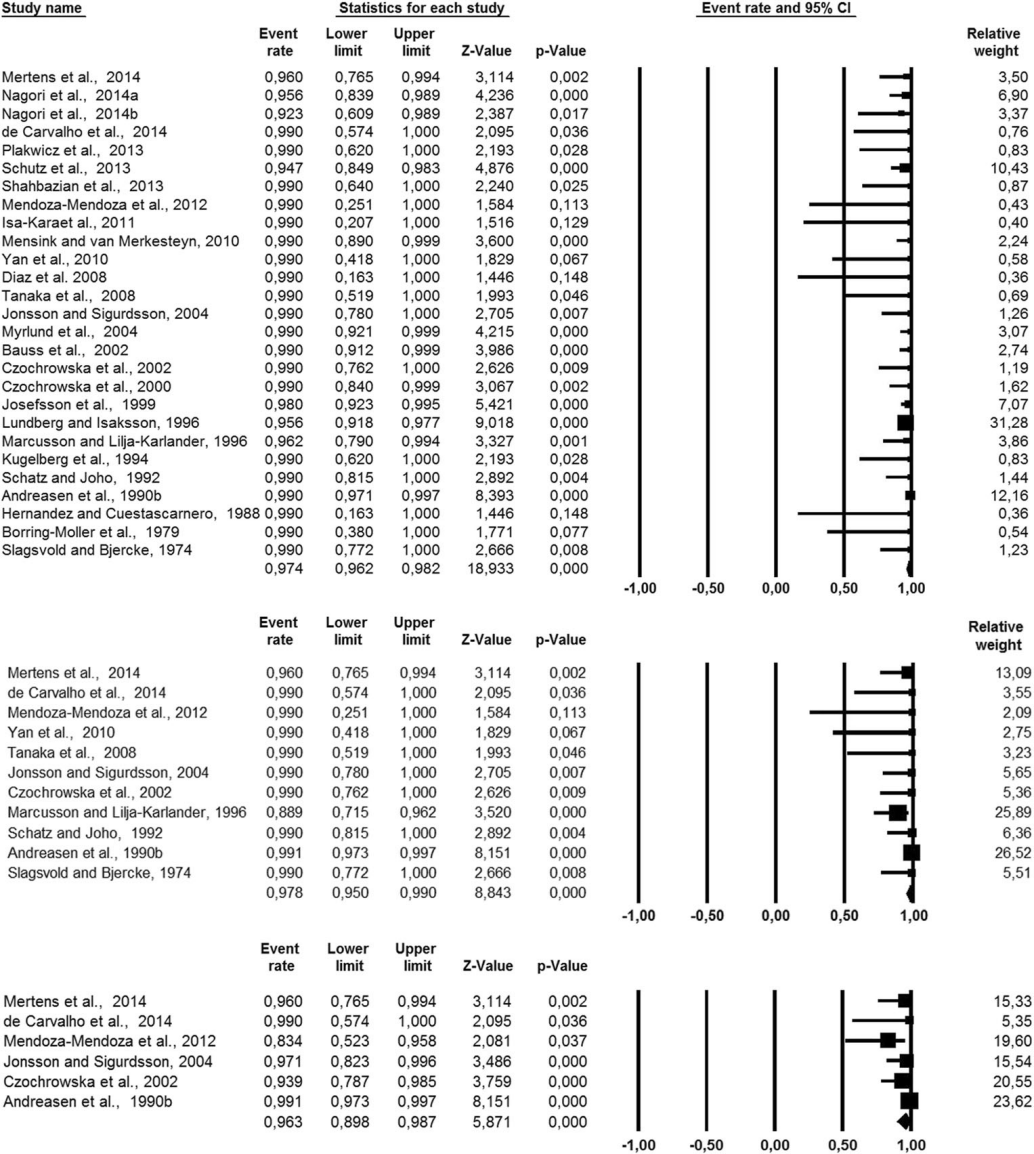
Meta-analysis of 1-, 5-, and 10-year survival rates of autotransplanted teeth in descending order

The survival rate after 1 year was reported in 26 articles with the average weighted survival rate of 97.4% (95% CI, 96.2–98.2%) (Fig. 2). No heterogeneity was found across these studies (*Q* = 13.66; *p* = 0.98; *I*^2^ = 0.0%). The survival rate after 5 years was reported in 11 articles with the average weighted survival rate of 97.8% (95% CI, 95.0–99.0%) (Fig. 2). The data on 5-year survival showed 19.6% heterogeneity (*Q* = 12.4; *p* = 0.26), which can be considered low. The survival rate after 10 years was reported in six articles with the average weighted survival rate of 96.3% (95% CI, 89.8–98.7%) (Fig. 2). The heterogeneity was 56.8%, which can be considered substantial (*Q* = 11.6; *p* = 0.04). The weighted estimated survival rate per year was 98.2% (95% CI, 96.4–99.1%) (Tables 2 and 3). No heterogeneity was found (*Q* = 6.2; *p* = 0.99; *I*^2^ = 0.0%).

**Table 2:** Rate of survival, success, and complications of the studies included

Author	Overall survival (%)	1-Year survival (%)	5-Year survival (%)	10-Year survival (%)	Success (%)	Ankylosis (%)	Root resorption (%)	Pulp necrosis (%)
Mertens et al. [31]	96	96	96	96	61.1	14.3	22.2	16.7
Nagori et al. [3]	95.6	95.6	–	–	86.7	–	11.1	2.2
Nagori et al. [4]	92.3	92.3	–	–	92.3	–	–	7.7
de Carvalho et al. [37]	75	100	100	100	–	–	–	–
Plakwicz et al. [5]	100	100	–	–	91.3	4.3	–	0
Schütz et al. [6]	94.7	94.7	–	–	94.7	0	–	3.5
Shahbazian et al. [33]	100	100	–	–	91.7	5	0	5
Mendoza-Mendoza et al. [7]	83.3	100	100	83.3	80	–	16.7	16.7
Isa-Kara et al. [8]	100	100	–	–	100	0	–	0
Vilhjálmsson et al. [9]	–	–	–	–	84.6	–	15.4	0
Gonnissen et al. [10]	–	–	–	–	70.6	–	–	–
Mensink and van Merkesteyn [11]	100	100	–	–	–	4.8	0	3.2
Yan et al. [12]	100	100	100	–	100	–	0	12.5
Díaz et al. [13]	100	100	–	–	–	0	10	40
Tanaka et al. [45]	100	100	100	–	100	–	–	–
Jonsson and Sigurdsson [14]	97.1	100	100	97.1	91.9	0	5.7	34.3
Myrlund et al. [46]	98.6	100	–	–	90.5	–	–	–
Bauss et al. [15]	100	100	–	–	84.2	5.3	–	9.2
Czochrowska et al. [16]	90.9	100	100	93.9	78.8	12.1	–	–
Czochrowska et al. [2]	100	100	–	–	93	2.2	4.4	–
Josefsson et al. [17]	98	98	–	–	91.9	3	–	–
Lundberg and Isaksson [18]	95.6	95.6	–	–	94.1	2.9	0.5	3.4
Marcusson and Lilja-Karlander [19]	85.2	96.2	88.9	–	–	0	6.5	0
Kugelberg et al. [20]	100	100	–	–	95.7	0	0	–
Schatz and Joho [21]	100	100	100	–	92.5	–	3.3	7.5
Kristerson and Lagerstrom [22]	–	–	–	–	90.2	–	–	–
Andreasen et al. [30]	99	100	99.1	99.1	–	–	–	7.4
Andreasen et al. [35]	–	–	–	–	–	3.6	3.3	–
Hernandez and Cuestascarnero [23]	100	100	–	–	100	0	0	–
Kristerson [24]	95.4	–	–	–	–	6.9	3.4	11.5
Borring-Møller et al. [25]	100	100	–	–	–	0	0	–
Slagsvold and Bjercke [1]	100	100	100	–	–	–	–	–

**Table 3:** Annual estimated weighted survival rates, success rates, ankylosis rates, root resorption rates, and necrotic pulp rates obtained from meta-analysis

Survival rate	
Overall (CI 95%)	98.2% (96.4–99.1%)
Premolar donor teeth (CI 95%) Molar donor teeth (CI 95%)	98.4% (96.3–99.4%) 97.2% (93.9–98.8%)^a^
The maxilla as recipient site (CI 95%)	a
The mandible as recipient site (CI 95%)	98.1% (86.7–99.7%)
The incisor region as recipient site (CI 95%)	a
The premolar region as recipient site (CI 95%)	98.6% (95.4–99.6%)
The molar region as recipient site (CI 95%)	97.3% (93.6–98.9%)
Success rate	
Overall (CI 95%)	98.6% (94.8–97.8%)
Canine donor teeth (CI 95%)	97.7% (73.6–99.8%)
Premolar donor teeth (CI 95%) Molar donor teeth (CI 95%)	98.1% (95.5–99.2%) 95.5% (92.0–97.5%)
The maxilla as recipient site (CI 95%)	98.5% (94.5–99.6%)
The mandible as recipient site (CI 95%)	97.3% (92.7–99.1%)
The incisor region as recipient site (CI 95%)	98.5% (93.8–99.7%)
The canine region as recipient site (CI 95%)	97.7% (73.6–99.8%)
The premolar region as recipient site (CI 95%)	97.8% (93.6–99.3%)
The molar region as recipient site (CI 95%)	95.1% (90.8–97.4%)
Ankylosis rate	
Overall (CI 95%)	2.0% (1.1–3.7%)
Premolar donor teeth (CI 95%) Molar donor teeth (CI 95%)	1.9% (0.8–4.7%) 2.2% (0.7–6.3%)
Root resorption rate	
Overall (CI 95%)	2.9% (1.5–5.5%)
Premolar donor teeth (CI 95%) Molar donor teeth (CI 95%)	1.5% (0.5–4.7%) 5.0% (2.1–11.7%)
Pulp necrosis rate	
Overall (CI 95%)	3.3% (1.9–5.6%)
Premolar donor teeth (CI 95%) Molar donor teeth (CI 95%)	4.4% (2.0–9.3%) 2.5% (1.0–5.9%)

Articles included are different for each meta-analysis and can be found in Appendix C

^a^It was not possible to conduct a meta-analysis because all articles had a survival rate of 100%

### Success rate

Twenty-three articles reported the success rate. The definition of success rate varied to a high degree between the included articles (see “Discussion”). The weighted estimated yearly success rate was 96.6% (95% CI, 94.8–97.8) (Table 3). No heterogeneity was found (*Q* = 8.24; *p* = 0.99; *I*^2^ = 0.0%).

### Complication rates

The weighted estimated ankylosis, root resorption, and pulp necrosis rates per year were 2.0% (95% CI, 1.1–3.7%), 2.9% (95% CI, 1.5–5.5%), and 3.3% (95% CI, 1.9–5.6%), respectively (Table 3).

## Secondary outcomes

### Donor tooth type

Two articles reported the survival and success rates of different donor teeth [18–22] with no difference detected. Most articles used one specific type of donor tooth (Table 1). No meta-analysis could be conducted for incisors, since autotransplantation of incisors is less common and the outcomes of interest were not specified in the five articles reporting the transplantation of incisors [9, 16, 20, 22, 37]. Only one article [10] reported the transplantation of canines and the weighted estimated success rate was 97.7% per year (95% CI, 73.6–99.8%). The survival and complication rates were not reported. The yearly weighted estimated success rate, survival rate, and the complication rates were calculated for the premolars and molars (Table 3).

### Recipient site

The yearly weighted estimated survival and success rates were calculated for the different recipient sites (Table 3, Appendix D).

#### Maxilla

All articles reported a 100% survival during follow-up after a mean follow-up period of 45.5 months [2, 6, 7, 13, 20, 22], and therefore no meta-analysis was conducted. The weighted estimated success rate per year was 98.5% (95% CI, 94.5–99.6%).

#### Mandible

The weighted estimated survival rate for the mandible as recipient site was 98.1% per year (95% CI, 86.7–99.7%) and the weighted estimated success rate was 97.3% (95% CI, 92.7–99.1%).

#### Incisor region

All studies reported a 100% survival rate during the follow-up (mean 61.6 months) for teeth transplanted to the incisor region. The weighted estimated success rate per year was 98.5% (95% CI, 93.8–99.7%).

#### Canine region

Only the success rate was provided in the article reporting on the canine region as a recipient site. The weighted estimated success rate per year was 97.7% (95% CI, 64–100%).

#### Premolar region

The weighted estimated survival rate per year was 98.6% (95% CI, 95.4–99.6%) and the success rate per year was 97.8% (95% CI, 93.6–99.3%).

#### Molar region

The weighted estimated survival rate per year was 97.3% (95% CI, 1.1–6.4%) and the success rate per year was 95.1% (95% CI, 90.8–97.4%).

### Root development

The majority of the transplants exhibited a 2 to 4 stage of root development (Table 1). Four articles reported the success and survival rates in relation to the stage of root development [7, 9, 22, 24]. The survival rate for the teeth transplanted for each stage was as follows: stage 1, 100% [24]; stage 2, 100% [7, 24]; stage 3, 85.7% (71.4–100%) [7, 9, 24]; stage 4, 93.8% (88.9–100%) [7, 9, 24]; and stage 5, 50% [24]. The success rates were for stage 1, 100% [22]; stage 2, 88.9% (85.7–100%) [7, 22]; stage 3, 87.5% (71.4–100%) [7, 9, 22]; stage 4, 90% (0–100%) [9, 22]; and stage 5, 66.7% (55.6–100%) [9, 22]. The number of teeth per stage was limited (survival median *n* = 8, success median *n* = 7). Therefore, no valid conclusions with regard to the effect of root development on the success rate of autotransplantation could be drawn.

### Surgical protocol and orthodontics

Most articles described the use of sutures or wires as a stabilization method (Table 1), with the latter applied in case of insufficient stability [3, 4, 12, 15, 18]. Conflicting results regarding the influence of stabilization technique on the success of autotransplantation were reported [3, 15]. No study provided information about the effect of splinting duration on the survival or success rates.

Orthodontics was applied in 15 studies as part of the treatment plan, none of which assessed the influence of orthodontic interventions on the survival or success rates of the transplanted teeth.

Systemic prophylactic antibiotics were prescribed pre- or postoperatively in 21 articles. In two studies, antibiotics were either not routinely used [14] or not used at all [16]. The other articles did not report about the use of antibiotics.

To summarize, the information in the articles was insufficient with respect to the effect of the stabilization method and duration, the orthodontic procedure, and the antibiotic regimen on the survival and success rates. Therefore, it was not possible to conduct a meta-analysis on these aspects.

## Discussion

This is the first systematic review and meta-analysis on the autotransplantation of teeth with incomplete root formation, using both survival and success rates as primary outcome parameters, focusing on long-term outcome and using elaborate statistical methodology to correct intrinsic heterogeneity among the included studies.

Using strict inclusion and exclusion criteria, 32 prospective and retrospective articles were included in the present review. High survival and success rates were recorded (up to 10 years) with relatively low complication rates.

The survival rates after 1, 5, and 10 years were respectively 97.4% (95% CI, 96.2–98.2%), 97.8% (95% CI, 95.0–99.0%), and 96.3% (95% CI, 89.8–98.7%). It is remarkable that the survival rates remain equally high during a follow-up period of 10 years and that most failures were observed in the first year. In other words, autotransplanted teeth that have survived after 1 year indicate a favorable prognosis for a longer period of survival of up to 10 years. There is also a chance that reporting and publication bias are part of the explanation. The high survival rates reported are in line with those of the literature. High survival (81–98.2%) was reported after 1 to > 6 years follow-up [32, 38, 39, 48].

Due to the wide range of the reported follow-up (1–26.4 years), the weighted estimated success rate per year (96.6%; 95% CI, 97.8–94.8%) was calculated. The definition of success rate varied among studies. Some authors considered a case successful if the tooth retained its vitality [5, 8], while others considered a case successful if a successful endodontic treatment was conducted after the development of pulp necrosis [9, 10, 22]. The most frequent success variables were the absence of progressive root resorption [5, 8–10, 14, 16–18, 20, 22–33], ankylosis [2, 6, 8, 15, 17, 20, 33, 45, 46], mobility [3, 4, 6, 8, 15, 23, 33, 45], pathologically increased probing depths [3, 4, 6, 10, 15, 23, 33], pulpal or apical inflammation [6, 8–10, 15, 18, 22], and crown-to-root ratio greater than 1 [5, 14, 16, 33, 45, 46]. Other authors defined success on the basis of radiographic signs of a normal periodontal ligament space and lamina dura [3, 4, 6, 9, 12, 23] and tooth presence at follow-up [10, 15, 17, 18, 45]. Because it was not possible to compensate for the differences in the definition of success, we included all studies that conform to our definition of success (see “Data extraction”). A more precise definition of success including clinically meaningful outcomes should be proposed as a guideline for future studies.

Ankylosis, root resorption, and pulp necrosis are the most commonly reported complication parameters. It has been previously assumed that ankylosis, if present, can be diagnosed within 1 year after transplantation [35]. A later study reported that the detection of root resorption may take up to 3 years [49]. Interestingly, we observed the highest ankylosis rates in studies with the longest follow-up [16, 31]. This suggests that ankylosis can become apparent years after the transplantation of the tooth. Progressive root resorption due to a damaged periodontal ligament or pulp infection has been radiographically observed 1–2 months after transplantation [34, 36]. In the present review, the reported root resorption rate ranged from 0 to 22.2%, with an estimated weighted rate of 2.9%, which is comparable with that of a previous report on transplantation of teeth with complete root formation (2.1%) [39]. The reported pulp necrosis rate varied even more (0–40%), and the difference in root development can be the reason for the variation [36]. However, the presence of pulp necrosis does not necessarily imply tooth failure or non-success, especially when endodontic treatment is conducted subsequently as was performed in most studies.

Molars showed overall less favorable results than premolars, which is in line with the literature [30, 32, 39]. The annual survival rate of premolars was higher for premolars in comparison to that for molars (98.4 vs. 97.2%). The annual success rates were also more favorable for premolars (98.1%) in comparison to those for molars (95.5%). Though similar, ankylosis rates were observed (2.2 vs. 1.9%, root resorption rates were higher in molars (5.0 vs. 1.5%). Premolars showed only less favorable outcome regarding pulp necrosis (4.4 vs. 2.5%). The more favorable results in premolars may be explained by factors such as the number of roots and the position in the jaw, which makes atraumatic removal and preservation of the periodontal ligament of the donor teeth easier [35]. Other factors could be the higher age of the patients and the difference in indications for transplantation. Since the articles are different in each group analyzed (Appendix D), the results must be interpreted with caution.

The most favorable results were found for transplantation to the region of incisors (annual success rate 98.5%), followed by premolars (97.8%), canines (97.7%), and molars (95.1%). More favorable outcomes were found in the maxilla (annual success rate 98.5%) compared to those in the mandible (97.3%) in accordance with a previous study [32]. Those small differences can partly be explained by the difference in donor tooth transplanted. Several combinations of tooth donor recipient sites were reported. Incisors were exclusively transplanted to incisor recipient sites [16, 20]; canines to canine sites [10] and only in case of trauma to incisor sites [20]. Premolars were transplanted to a wide range of recipient sites ranging from incisor [2, 5, 7, 13, 16, 20, 22, 45], canine [16, 45], and premolar recipient sites [5, 11, 14, 16, 17, 30, 35, 45, 50]. Molars were solely transplanted to premolar [6, 15, 17] and molar sites [3, 4, 6, 8, 12, 15, 23, 25].

With respect to the influence of the stage of root formation on the survival and success rates, we found insufficient evidence to favor transplantation of teeth between stages 2 and 3, as previously reported [7, 24, 32, 34, 38], since most articles did not report the outcomes for the development stage separately.

No meta-analyses could be performed on the influence of the use of prophylactic antibiotics, the stabilization method and duration, and orthodontic treatment on the survival and success rates, because of insufficient information on these parameters. Results from the present review cannot confirm or reject the recommendation in the literature for the use of prophylactic antibiotics [39, 51] or sutures as stabilization method [15, 28, 35]. Tooth transplantation is often a part of an orthodontic treatment plan, but the question remains whether orthodontics force affects the success, survival, or complication rates of a transplanted teeth compared to those without orthodontic intervention.

The majority of studies followed the protocol of Andreasen et al. [44] or one alike, meaning, surgical planning of the autotransplantation was based on periapical or panoramic radiographs. Recently, cone beam computed tomography (CBCT) to assist surgical planning was introduced [33]. Most articles reporting on CBCT planning and the use of a 3D tooth replica are case reports, and only one article met the criteria and was included in this review. This article showed encouraging results such as shorter and less invasive surgery and low failures [33]. However, more research needs to be done with larger power and proper control to conclude if the application of CBCT in planning further improves the outcomes of tooth autotransplantation in comparison to the conventional approach.

### Limitations

No randomized controlled clinical trials have been published on autotransplantation of teeth, so only prospective and retrospective cohort studies and case series were included in this review. Though RCTs would be preferred, the nature of tooth autotransplantation makes it practically impossible or even unethical to perform single- or double-blind studies to assess the influence of root formation stage, receptor site, or donor tooth, et cetera. Currently, only retrospective studies were available to examine the long-term follow-up results. Prospective well-designed studies are necessary in the future to confirm the outcomes obtained from retrospective studies. The follow-up period varied considerably in the included articles (12–317 months). Only studies with a mean follow-up of at least 1 year were included. Studies reporting follow-up of less than a year [5, 6, 9, 13, 18, 25, 37] likely show an underestimation of the complications and an overestimation of the survival and success. To minimize the chance of publication bias and effect of the studies with small sample size, the present review only included articles with at least 10 autotransplanted teeth resulting in a median sample size of 33. Nevertheless, it has to be acknowledged that due to the relatively small sample size of most studies and insufficient studies of high quality, the results from the meta-analysis are of limited level of evidence, and therefore must be interpreted with caution.

## Conclusions

Within the limitations of this review, we may conclude that autotransplantation of teeth with incomplete root formation could be considered as a treatment option for tooth replacement. One-, 5-, and 10-year survival and success rates were high (> 90%) and complications in terms of ankylosis, root resorption, and pulp necrosis were very low. Premolars were slightly preferred over molars as donor teeth. Existing evidence on prognostic factors such as stage of root formation, postsurgical stabilization methods, and orthodontic treatment is insubstantial to merit a firm conclusion.

Results from the present review put forward a number of recommendations for future research: (1) randomized controlled trials on specific aspects (CBCT planning, stabilization methods, timing of orthodontic load) with adequate power analysis; (2) prospective studies with longer follow-up to better understand and identify the prognostic factors for survival and success; (3) a general consensus on the definition of “success.”

## Electronic supplementary material

ESM 1 (DOC 62 kb)

ESM 2 (DOCX 14 kb)

ESM 3 (DOCX 22 kb)

ESM 4 (DOCX 16 kb)
