# Supplementary material for: Autotransplantation of teeth with incomplete root formation: a systematic review and meta-analysis
Source: Clin Oral Investig. 2018 Mar 10;22(4):1613–24. doi: 10.1007/s00784-018-2408-z (PMC5906482; doi:10.1007/s00784-018-2408-z)
Supplement: Supplementary file 2 — (DOCX 14 kb) [file 784_2018_2408_MOESM2_ESM.docx]

**Appendix B.** Search protocol for PubMed

| PubMed | ((autotransplant[tw] OR autotransplantants[tw] OR autotransplantation[tw] OR autotransplantations[tw] OR autotransplanted[tw] OR autotransplantions[tw] OR autotransplantion[tw] OR autotransplants[tw]) OR (transplant[tw] OR transplantated[tw] OR transplantates[tw] OR transplantation[tw] OR transplantations[tw] OR transplanted [tw] OR transplants [tw])) AND ("tooth"[MeSH Terms] OR teeth[tw] OR tooth[tw] OR premolar[tw] OR premolars[tw] OR bicuspid[tw] OR bicuspids[tw] OR incisor[tw] OR incisors[tw] OR cuspid[tw] OR cuspidate[tw] OR cuspids[tw] OR molar[tw] OR molars[tw])) |
| --- | --- |
