# Supplementary material for: Autotransplantation of teeth with incomplete root formation: a systematic review and meta-analysis
Source: Clin Oral Investig. 2018 Mar 10;22(4):1613–24. doi: 10.1007/s00784-018-2408-z (PMC5906482; doi:10.1007/s00784-018-2408-z)
Supplement: Supplementary file 3 — (DOCX 22 kb) [file 784_2018_2408_MOESM3_ESM.docx]

|  | **Selection** | 1. Representativeness of the exposed cohort | 1. Selection of the non-exposed cohort | 1. Ascertainment of exposure | 1. Demonstration that outcome of interest was not present at start of study | **Comparability** | 1. Comparability of cohorts on the basis of the design or analysis | **Outcome** | 1. Assessment of outcome | 1. Was follow-up long enough for outcomes to occur | 1. Adequacy of follow up of cohorts |
| --- | --- | --- | --- | --- | --- | --- | --- | --- | --- | --- | --- |
| Mertens et al. [31] |  | ★ | NA | ★ | ☆ |  | ★☆ |  | ☆ | ★ | ☆ |
| Nagori et al. [3] |  | ★ | NA | ★ | ★ |  | ★☆ |  | ☆ | ★ | ★ |
| Nagori et al. [4] |  | ★ | NA | ★ | ☆ |  | ★☆ |  | ☆ | ★ | ★ |
| de Carvalho et al. [36] |  | ★ | NA | ★ | ★ |  | ★☆ |  | ☆ | ★ | ★ |
| Plakwicz et al. [5] |  | ★ | NA | ★ | ★ |  | ★★ |  | ☆ | ★ | ★ |
| Schütz et al. [6] |  | ★ | NA | ★ | ☆ |  | ★☆ |  | ☆ | ★ | ★ |
| Shahbazian et al. [33] |  | ★ | NA | ★ | ★ |  | ★☆ |  | ★ | ★ | ★ |
| Mendoza-Mendoza et al. [7] |  | ★ | NA | ★ | ☆ |  | ★☆ |  | ☆ | ★ | ☆ |
| Isa-Kara et al. [8] |  | ★ | NA | ★ | ★ |  | ★☆ |  | ☆ | ★ | ★ |
| Vilhjálmsson et al. [9] |  | ★ | NA | ★ | ☆ |  | ★★ |  | ☆ | ★ | ★ |
| Gonnissen et al. [10] |  | ★ | NA | ★ | ☆ |  | ★☆ |  | ☆ | ★ | ☆ |
| Mensink and van Merkesteyn [11] |  | ★ | NA | ★ | ☆ |  | ★☆ |  | ☆ | ★ | ★ |
| Yan et al. [12] |  | ★ | NA | ★ | ★ |  | ★☆ |  | ☆ | ★ | ★ |
| Díaz et al. [13] |  | ★ | NA | ★ | ★ |  | ★☆ |  | ☆ | ★ | ★ |
| Tanaka et al. [46] |  | ★ | NA | ★ | ☆ |  | ★☆ |  | ☆ | ★ | ★ |
| Jonsson and Sigurdsson [14] |  | ★ | NA | ★ | ☆ |  | ★☆ |  | ☆ | ★ | ★ |
| Myrlund et al. [48] |  | ★ | NA | ★ | ☆ |  | ★★ |  | ☆ | ★ | ☆ |
| Bauss et al. [15] |  | ★ | NA | ★ | ★ |  | ★★ |  | ☆ | ★ | ★ |
| Czochrowska et al. [16] |  | ★ | NA | ★ | ☆ |  | ★☆ |  | ☆ | ★ | ☆ |
| Czochrowska et al. [2] |  | ★ | NA | ★ | ☆ |  | ★☆ |  | ☆ | ★ | ☆ |
| Josefsson et al. [17] |  | ★ | NA | ★ | ☆ |  | ★☆ |  | ☆ | ★ | ★ |
| Lundberg and Isaksson [18] |  | ★ | NA | ★ | ☆ |  | ★☆ |  | ☆ | ★ | ★ |
| Marcusson and Lilja-Karlander [19] |  | ★ | NA | ★ | ☆ |  | ★☆ |  | ☆ | ★ | ★ |
| Kugelberg et al. [20] |  | ★ | NA | ★ | ☆ |  | ★☆ |  | ☆ | ★ | ★ |
| Schatz and Joho [21] |  | ★ | NA | ★ | ★ |  | ★★ |  | ☆ | ★ | ★ |
| Kristerson and Lagerstrom [22] |  | ★ | NA | ★ | ☆ |  | ★☆ |  | ☆ | ★ | ★ |
| Andreasen et al. [30] |  | ★ | NA | ★ | ★ |  | ★☆ |  | ☆ | ★ | ★ |
| Andreasen et al. [34] |  | ★ | NA | ★ | ★ |  | ★☆ |  | ☆ | ★ | ★ |
| Hernandez and Cuestascarnero [23] |  | ★ | NA | ★ | ★ |  | ★☆ |  | ☆ | ★ | ★ |
| Kristerson [24] |  | ★ | NA | ★ | ★ |  | ★★ |  | ☆ | ★ | ★ |
| Borring-Møller et al. [25] |  | ★ | NA | ★ | ★ |  | ★☆ |  | ☆ | ★ | ★ |
| Slagsvold and Bjercke [1] |  | ★ | NA | ★ | ☆ |  | ★☆ |  | ☆ | ★ | ☆ |

**Appendix C** NOS-scale
