# Supplementary material for: Autotransplantation of teeth with incomplete root formation: a systematic review and meta-analysis
Source: Clin Oral Investig. 2018 Mar 10;22(4):1613–24. doi: 10.1007/s00784-018-2408-z (PMC5906482; doi:10.1007/s00784-018-2408-z)
Supplement: Supplementary file 4 — (DOCX 16 kb) [file 784_2018_2408_MOESM4_ESM.docx]

**Appendix D.** Studies included in the meta-analyses

| Meta-analysis | Articles included |
| --- | --- |
| Overall failure rate in terms of survival | 1-8, 11-17, 20-21, 23-25, 33, 36, 46, 48 |
| Failure rate in terms of survival of premolar donor teeth | 2, 5, 7, 11, 13-14, 20, 24, 46- 48 |
| Failure rate in terms of survival of molar donor teeth | 3-4, 6, 8, 12, 15, 23, 25 |
| Failure rate in terms of survival of the maxilla as recipient site | 2, 6-7, 13, 20 |
| Failure rate in terms of survival of the mandible as recipient site | 6, 17 |
| Failure rate in terms of survival of the incisor region as recipient site | 2, 7, 13, 20 |
| Failure rate in terms of survival of the premolar region as recipient site | 6, 11, 14, 17, 47 |
| Failure rate in terms of survival of the molar region as recipient site | 3, 4, 6, 8, 12, 15, 23, 25 |
| Overall failure rate in terms of success | 2-10, 12, 14-16, 20-23, 33, 46, 48 |
| Failure rate in terms of success of canine donor teeth | 10 |
| Failure rate in terms of success of premolar donor teeth | 1, 2, 5, 7, 14, 20, 22, 46, 48 |
| Failure rate in terms of success of molar donor teeth | 3-4, 6, 8, 12, 15, 22-23, 25 |
| Failure rate in terms of success of the maxilla as recipient site | 2, 6-7, 20, 22 |
| Failure rate in terms of success of the mandible as recipient site | 6, 17 |
| Failure rate in terms of success of the incisor region as recipient site | 2, 7, 20, 22 |
| Failure rate in terms of success of the canine region as recipient site | 10 |
| Failure rate in terms of success of the premolar region as recipient site | 6, 14, 17 |
| Failure rate in terms of success of the molar region as recipient site | 3, 4, 6, 8, 12, 15, 23 |
| Overall ankylosis rate | 2, 5-6, 8, 11, 13-17, 20, 23-25, 33 |
| Ankylosis rate of premolar donor teeth | 5, 10-11, 13-14, 20, 24 |
| Ankylosis rate of molar donor teeth | 6, 8, 15, 23, 25 |
| Overall root resorption rate | 2-3, 7, 9, 11-14, 20-21, 23-25, 33 |
| Root resorption rate of premolar donor teeth | 7, 10-11, 13-14, 20, 24 |
| Root resorption rate of molar donor teeth | 3, 8, 12, 23, 25 |
| Overall pulp necrosis rate | 2, 3, 5-9, 11-15, 21, 24, 33 |
| Pulp necrosis rate of premolar donor teeth | 5, 7, 11, 13-14, 24 |
| Pulp necrosis rate of molar donor teeth | 3-4, 6, 8, 12, 15 |
